# Numerical Modelling of Hybrid Polymer Composite Frame for Selected Construction Parts and Experimental Validation of Mechanical Properties

**DOI:** 10.3390/polym17020168

**Published:** 2025-01-11

**Authors:** Tegginamath Akshat, Michal Petru, Rajesh Kumar Mishra

**Affiliations:** 1 Department of Machine Parts and Mechanism, Faculty of Mechanical Engineering, Technical University of Liberec, Studentská 1402/2, 46117 Liberec, Czech Republic; akshattm93@gmail.com (T.A.); michal.petru@tul.cz (M.P.); 2 Department of Material Science and Manufacturing Technology, Faculty of Engineering, Czech University of Life Sciences Prague, Kamycka 129, 16500 Prague, Czech Republic

**Keywords:** hybrid composite, tensile test, mechanical properties, finite element modeling (FEM), interlaminar shear strength (ILSS), computed tomography

## Abstract

This article is a numerical and experimental study of the mechanical properties of different glass, flax and hybrid composites. By utilizing hybrid composites consisting of natural fibers, the aim is to eventually reduce the percentage usage of synthetic or man-made fibers in composites and obtain similar levels of mechanical properties that are offered by composites using synthetic fibers. This in turn would lead to greener composites being utilized. The advantage of which would be the presence of similar mechanical properties as those of composites made from synthetic fibers along with a reduction in the overall weight of components, leading to much more eco-friendly vehicles. Finite element simulations (FEM) of mechanical properties were performed using ANSYS. The FEM simulations and analysis were performed using standards as required. Subsequently, actual beams/frames with a defined geometry were fabricated for applications in automotive body construction. The tensile performance of such frames was also simulated using ANSYS-based models and was experimentally verified. A correlation with the results of the FEM simulations of mechanical properties was established. The maximum tensile strength of 415 MPa was found for sample 1: G-E (glass–epoxy composite) and the minimum strength of 146 MPa was found for sample 2: F-G-E (G-4) (flax–glass–epoxy composite). The trends were similar, as obtained by simulation using ANSYS. A comparison of the results showed the accuracy of the numerical simulation and experimental specimens with a maximum error of about 8.05%. The experimental study of the tensile properties of polymer matrix composites was supplemented with interlaminar shear strength, and a high accuracy was found. Further, the maximum interlaminar shear strength (ILSS) of 18.5 MPa was observed for sample 1: G-E and the minimum ILSS of 17.0 MPa was observed for sample 2: F-G-E (G-4). The internal fractures were analyzed using a computer tomography analyzer (CTAn). Sample 2: F-G-E (G-4) showed significant interlaminar cracking, while sample 1: G-E showed fiber failure through the cross section rather than interlaminar failure. The results indicate a practical solution of a polymer composite frame as a replacement for existing heavier components in a car, thus helping towards weight reduction and fuel efficiency.

## 1. Introduction

Due to their light weight and the high stiffness characteristics exhibited by fiber-reinforced polymer composites, frames and other internal components in automobiles made traditionally from metals can be replaced with these composites. These composite materials are being used extensively in the transportation and aviation sector to achieve superior mechanical performance along with a reduction in weight. The increasing interest in a reduction in the environmental impact of materials over the last ten years has led to the development of composite materials which are sustainable and eco-friendly, where natural fibers are used as reinforcing materials [1,2,3]. The ability to customize material properties to satisfy various needs is one of the numerous benefits of composite materials in general. Void/gap condensation, yarn bending deformation, yarn cross section deformation and nesting are the five major mechanisms of compaction of the preforms. The mechanical properties of the composites are directly impacted by the ultimate microstructure, which is a result of compaction [4,5]. The mechanical properties of the composite are dependent on the mechanical properties of the yarn, and these depend on the cross section of the yarn. The properties of the interface and the geometrical arrangement of the phases dictate the macroscopic behavior of heterogenous fiber-reinforced polymer composites [6]. The mechanical properties of fiber-reinforced composites are also dictated by the yarn fiber volume ratio which can vary from 0.76 to 0.9, and for an ideal structure with unidirectional hexagonal packing, the value is 0.98 [7,8]. Some researchers have looked into the distribution of fibers in the yarn cross section and the fiber packing density of idealized yarn constructions [8,9,10]. Hence, it can be concluded that the mechanical properties of the composite are significantly impacted by the fiber volume fraction [8,11].

Hybrid fabric layers have been the focus of recent developments due to their versatile physical and structural attributes and application scope. These fabrics can be manufactured in various architectures, which offer a great deal of opportunity to modify the weight, physical and mechanical properties, and cost of the various products. Many of these hybrid structures can be produced on conventional weaving machines with slight modifications [10]. The combination of biofibers, e.g., sisal, flax, jute, etc., with industrial fibers in a hybrid construction is a further step toward sustainability and ecofriendly composite materials [11]. Cellulosic fibers are relatively weaker in terms of mechanical performance than industrial fibers, e.g., glass or carbon. The outer fabric layers can be composed of high-performance fibers. Such hybrid composites can replace existing components in cars, buildings, furniture, sports equipment, etc. Recently, a large amount of research has been conducted in the area of hybrid composites, where two or more reinforcements are used to reinforce a matrix material [12]. The primary objective of hybrid composites is to overcome the drawbacks of single-reinforcement composites by adding another type of reinforcement. The properties and sometimes the cost of reinforcement are optimized for the development of environmentally and economically sustainable composites for common applications. These hybrid composites have been tested for many applications, including wind turbine blades, automotive components and household items.

There are several components which can be fabricated and then assembled in cars [13,14,15]. The frames used in cars need to be ultralight and mechanically robust. Fiber-reinforced composite frames can be designed to replace the existing panels and frames in cars. High-performance yarns, e.g., kevlar, carbon, basalt or glass, can be used to design the frames, and they can be converted to composites by using thermoset resins. Performance can be predicted using advanced computational tools. Composite frame structures have been frequently designed as components of mechanical systems to resist lateral and gravitational loads. The manufacturing of high-quality composite frames depends primarily on accurate fiber winding on frames with different profiles and curved shapes [16,17,18,19,20].

Mesoscale finite element modeling of fiber-reinforced composites refers to the unit cell of the structure. It is a powerful tool for homogenizing mechanical properties, studying stress–strain fields inside a unit cell, determining damage initiation conditions for reinforced composites and simulating damage development and associated deterioration of the homogenized mechanical properties of composites.

Fibers from banana, jute hemp, sisal flax, etc., are commonly used in the manufacture of plant-based natural fiber-reinforced polymer composites [21,22]. When compared with the synthetic fibers, natural fibers offer advantages such as ease of machineability, zero toxicity, lower costs, a nonabrasive nature, biodegradability and a light weight [23,24,25,26,27,28]. Hemp, arrowroot, sisal, jute, kenaf, coir, pinecone and bamboo fibers have been used in natural fiber composites and their physical and mechanical properties have been investigated in detail [29,30,31,32,33,34]. The thermal and mechanical properties of natural fibers can be improved by the addition of another natural fiber/synthetic fiber in the hybrid bio composite.

Numerous studies have been carried out to determine the properties of various natural fiber-reinforced polymer composites, and sugar palm fiber was tested for use as a reinforcing material in hybrid composites. However, there is limited research on the modeling of natural fiber-based hybrid composites used in load bearing applications. The current research work is focused on the modeling and testing of composite plates reinforced with hybrid layers of glass and flax fabrics. The predicted results are compared with the experimental data obtained. In order to create an accurate model for the purposes of simulations, key material parameters used in the making of the composite plates were collected and entered while modeling the materials in ANSYS. Further, the interlaminar shear strength (ILSS), which indicates the fiber–matrix bonding at the inter layer spaces, was experimentally determined. The results of tensile tests were correlated with the ILSS. Computed tomography was used as a tool to determine the internal cracks in the tested samples by using X-rays. The overall findings of the computational models and the experimental results establish a foundation for new generation light weight construction materials for automotive components.

## 2. Materials and Methods

### 2.1. Materials

The properties of fiber and fabric materials used in this work are listed in Table 1.

#### Matrix/Resin

Two-component structural epoxy resin LH 288 with hardener H 282 from Havel Composites CZ s. r. o., Prague, Czech Republic was used as the resin. This matrix is characterized by a low viscosity of 500–900 mPa × s at 25 °C, which is important for ensuring sufficient wetting of fabrics by lamination. The density is 1100–1200 kg/m^3^ at 25 °C. The percentage of matrix used for impregnation was 55%.

### 2.2. Methods

#### 2.2.1. Modeling Software

SolidWorks 2024 was used for the three-dimensional modeling of woven structures and composites. An element was defined by eight nodes having three degrees of freedom at each node. Translations in the nodal x, y and z directions were considered. The element was assumed to have plasticity, creep, swelling, stress stiffening, large deflection and large strain capabilities. A reduced integration option with time control was used.

ANSYS 2024 was used for simulation of the composite models and prediction of mechanical performance. During the simulation for such multiple filament tows, the number of nodes and elements becomes extremely high, and the simulation time increases to more than 24 h. To optimize the processing time during the use of ANSYS, yarn/tow models were simplified into consolidated structures rather than individual filaments.

The approach of homogenization was used for the composites reinforced with 3 different types of S-glass woven fabrics on the outer layers and a plain-woven flax fabric sandwiched in the middle layer.

#### 2.2.2. Preparation of Samples

Samples of the composites were produced by hand lamination followed by vacuum impregnation. The manufacturing process was as follows:

(1) Spraying of the surface with a release agent before applying the first layer of fabric with separators.
(2) Use of epoxy resin on the S-glass fabric layer by hand lay-up.
(3) The addition of a flax fabric layer with resin/matrix. Layering of the upper S-glass fabric with resin/matrix with microparticles. A fiber volume fraction of 45% was used for all the samples.
(4) An auxiliary fabric was used to enable easy distribution of the resin and easy separation of the composite samples from the mold.
(5) Vacuum impregnation of the sandwich composite was performed under 600 bar pressure.
(6) The samples were cured for 24 h at a laboratory temperature of 22 ± 1 °C and a relative humidity of 41%.

In total, 30 samples were developed, which included control resin, nonhybrid composites of S-glass and flax, and three types of hybrid sandwich composites. All the samples were produced with an equal fiber volume fraction (0.45) and equal thickness (3 ± 0.1 mm) using 3 layers of fabric for comparison purposes.

#### 2.2.3. List of Samples

The samples developed are listed in Table 2.

#### 2.2.4. Experimental Testing of the Mechanical Properties

The experimental testing of the mechanical properties was carried out on a universal testing machine (LABTest 5.50ST; Labotech s.r.o., Opava, Czech Republic) with a scanning unit AST (type KAF 50 kN) and the evaluation software Test & Motion. The test speed was set at 2 mm/min. The tensile strength/maximum force (Fmax) and elongation at break were evaluated. The parameters of the tensile test are given below:

Test Standard: ASTM D 3039 [41];

Test Speed: 2 mm/min;

Pre-load: 10 N;

Tab length: 40 mm (on each side);

Gauge length: 150 mm.

**Figure 1:**
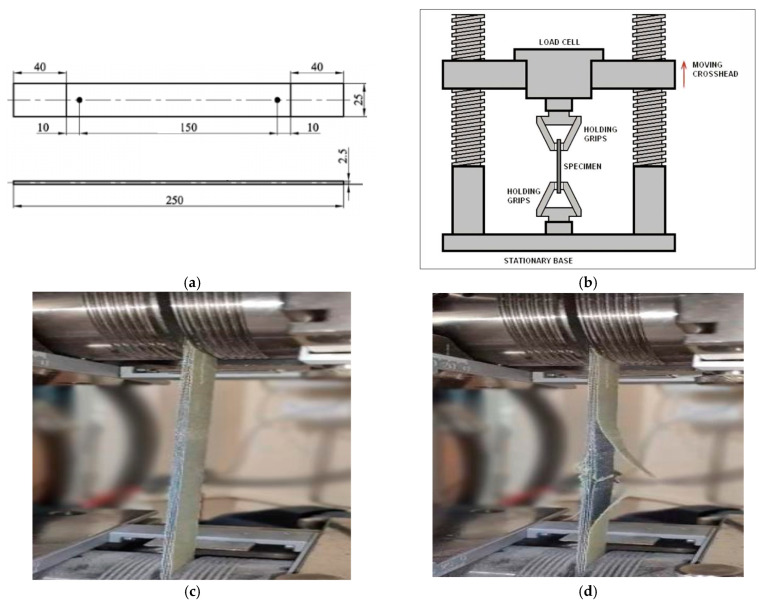
(**a**) ASTM standard for sample to be tested, (**b**) principle of tensile tester, (**c**) testing of the samples, (**d**) end of testing of the samples showing the breakage which occurs in the composite sample.

According to the ASTM D 3039 standards, the tensile testing of polymer matrix composite materials determines the in-plane tensile properties of the polymer matrix composite reinforced with fibers, and the standard also specifies that the length of the sample is 250 mm with a thickness of 2.5 mm and a width of 25 mm. The gauge length as specified by the sample is 150 mm with the tab length of each side of the sample being specified as 40 mm. The principle of the tensile testing is shown in Figure 1.

The experiments were repeated 20 times for each sample and the mean values were reported. Further, the statistical significance was determined so as to have a minimum coefficient of variation.

### 2.3. Interlaminar Shear Strength (ILSS) Measurement

**Figure 2:**
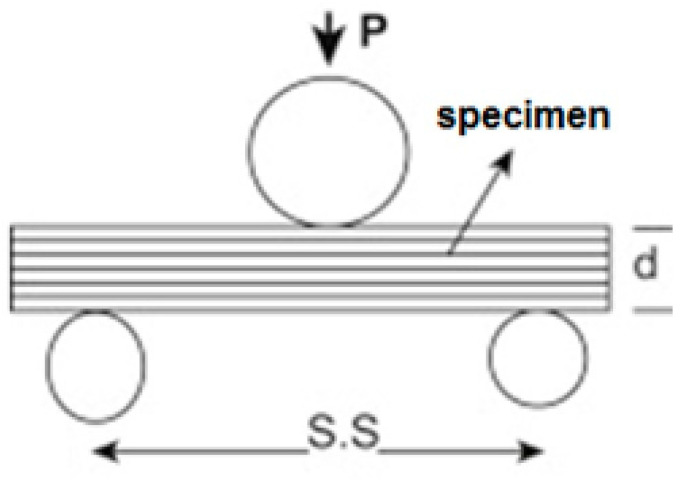
ILSS using short beam shear strength test.

The interlaminar shear strength of the samples was determined using a short beam shear strength test according to standard ASTM D 2344 [42]. It was used to evaluate the influence of fiber–matrix bonding on the ILSS at a laminate level but as a mesoscale characterization. It is a simple mode II transverse shear loading test that is meant to measure the quality of interfacial bonding. Figure 2 shows a schematic of the ILSS test in which the support span (S.S) length-to-thickness (h) ratio was 4:1. The test was carried out under a three-point monotonic flexural loading condition. For each sample, 10 measurements were carried out. The mean and standard deviation were calculated.

The interlaminar shear strength (ILSS) is given by Equation (1).

(1) $$\sigma_{s}=0.75P/bh$$

where $\sigma_{s}$ is ILSS, *P* is the maximum load, *b* = the width of specimen and *h* = the thickness of the sample.

### 2.4. Computed Tomography Analysis (CTAn)

CT-Analyser (CTAn) is an application for measuring quantitative parameters and constructing visual models from scanned 3D datasets obtained with SkyScan microCT instruments (Bruker, Billerica, MA, USA). The following parameters were used for CT analysis: pixel size 0.48 µm, voltage 50 kV. The quantitative measurement was made up of both densitometry (voxel attenuation coefficient or calibrated density) and morphometry, with the latter based on a segmented (black and white) image. CT-volume (“CTVol”) is an application for viewing and manipulating 3D surface rendered models from micro-CT scans. The 3D models are created in the program CT-analyser (“CTAn”). The purpose of these models is to produce visible and tangible aspects of the 3D structure of an object that has been imaged by micro-CT. Single and multiple models can be viewed. Multiple models can be made from the same scan of one object, for instance to visualize different material components of the object with differing x-ray opacities, using different visual properties such as color and transparency to make the components simultaneously visible.

### 2.5. Modeling Methodology

**Figure 3:**
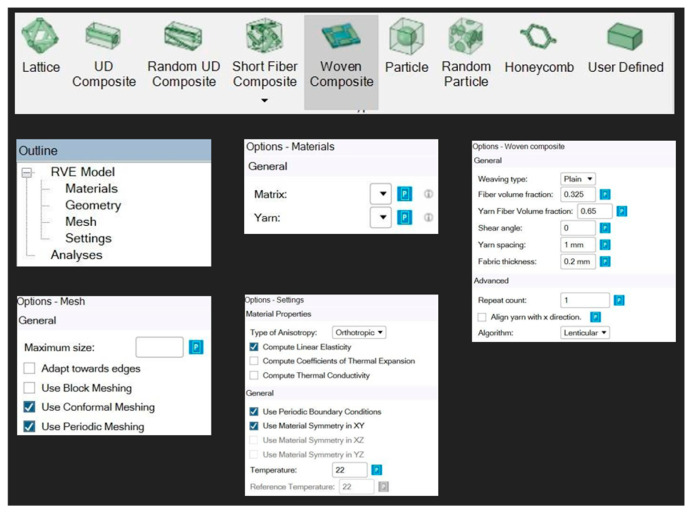
Parameters to be set while forming a fabric in Material Designer.

In order to create a composite with numerous layers of woven fabric in ANSYS, the woven fabrics must be created first. The option of woven composite was selected and details of the woven fabric (weave type, yarn volume fraction, shear angle, yarn spacing and fabric thickness) were entered, along with selection of the yarn type and the matrix. After which, the meshing parameters and other material properties like the orthotropic nature of the material were selected. The detailed scheme is shown in Figure 3.

#### 2.5.1. Dimensions of the Samples

The sample dimensions used for testing were length: 250 mm, width: 25 mm and height: 2.5 mm.

**Figure 4:**
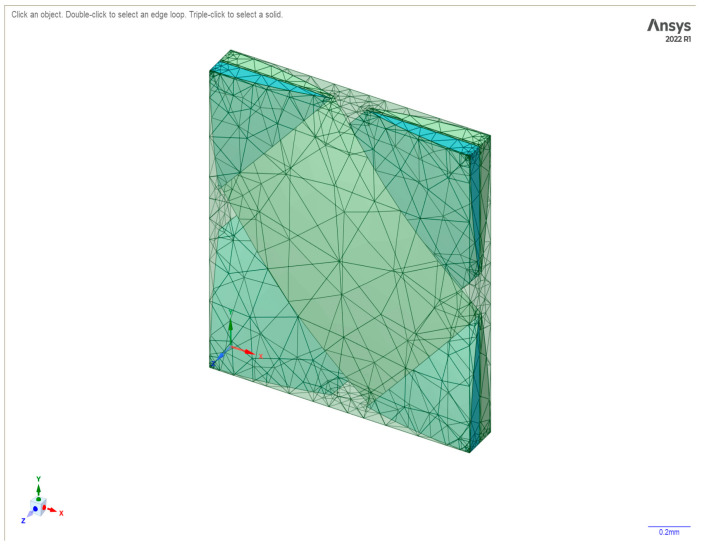
Composite fabric in Space Claim—Material Designer.

Figure 4 shows the meshing pattern in the unit cell of the woven fabric created in Material Designer in ANSYS after setting the various parameters as shown in Figure 3.

All samples were created in ANSYS following the same steps.

**Step 1:** Creating the composite fabric:

To create a composite fabric in Material Designer the following data have to be fed:

Weave type;

Yarn spacing;

Fiber volume fraction;

Yarn spacing;

Thickness.

The data which were fed defined the fabric and helped in creating the fabric virtually and a set of data were generated in Material Designer along with the generation of the fabric.

**Step 2:** The data that were generated in Material Designer were transferred to the Engineering Data module in Workbench.

**Step 3:** The data from the Engineering Data module were then transferred to the Engineering Data section of the ACP (Pre) module.

**Figure 5:**
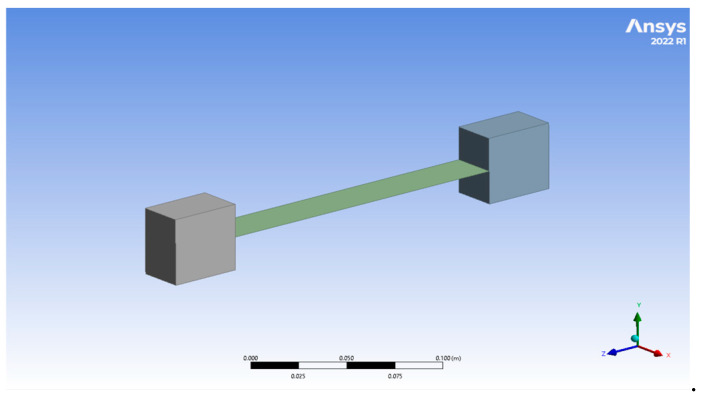
Imported geometry in Design Modeler.

**Step 4:** Using CAD (SolidWorks) software, version 2024, a geometry of the sample was created, which was then imported to Design Modeler in ANSYS. The geometry that was imported is shown in Figure 5.

**Step 5:** The geometry which was imported had the clamps and the sample. The “stiffness behavior” of the 2 clamps was defined as “rigid” and that of the sample was defined as “flexible”. Here, we also define the thickness of the sample as 2.5 mm.

**Figure 6:**
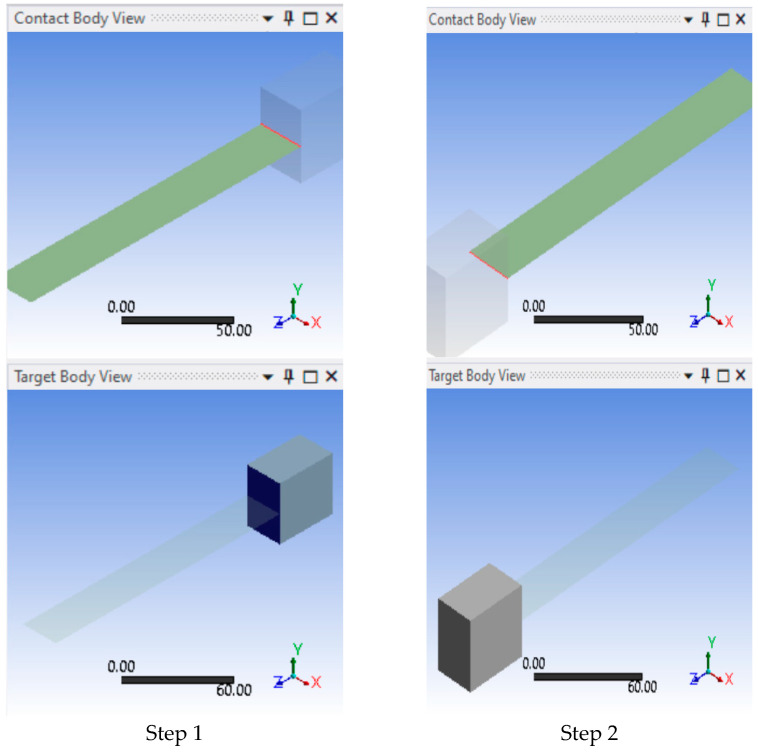
Inserting manual contact region.

**Step 6:** Since the imported geometry contained the clamps, as well as the sample, the contact region between the clamps and the sample had to be defined. Figure 6 shows the insertion of the manual contact regions between the sample and the clamp on either side of the sample in 2 steps.

**Figure 7:**
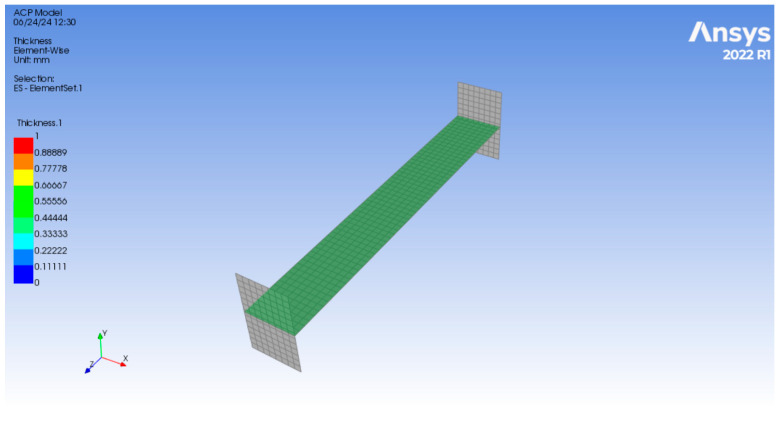
Generating the fabrics and defining the properties of the fabrics.

**Step 7:** We added the fabrics in the setup module in ACP (Ansys Composite PrePost) and defined the fabric properties for all the 8 fabric layers as shown in Figure 7.

Here, the thickness was defined as 0.3125 mm as the sample had 8 layers with a total thickness of 2.5 mm, and therefore, the thickness of one layer of fabric was given by (2.5/8), which is 0.3125 mm.

**Step 8:** The elements which were to be taken into consideration for testing were selected.

**Step 9:** Rosette and the Oriented Selection Set were defined and modeling ply was created with the appropriate properties.

**Step 10:** After creating the plies with the appropriate properties, we transferred the data from the ACP (Pre) module to the static structural module.

**Step 11:** In the static structure, the simulation was run to determine the stress values for the given sample, the results of which are given in Table 2, where the results obtained in Ansys are compared with the results obtained while testing the sample.

In the following steps, the samples are simulated to show the formation of a break in the sample when the load is applied.

**Step 12:** To simulate the breakage of the sample, data generated in ACP (Pre) module were transferred to the explicit dynamics module.

#### 2.5.2. Calculation of Percentage Extension

The general formula used in the calculation of the percentage extension is given as

Extension % = (Final length between the gauges − Initial length between the gauges)/(Initial length between the gauges) × 100

## 3. Results and Discussion

**Figure 8:**
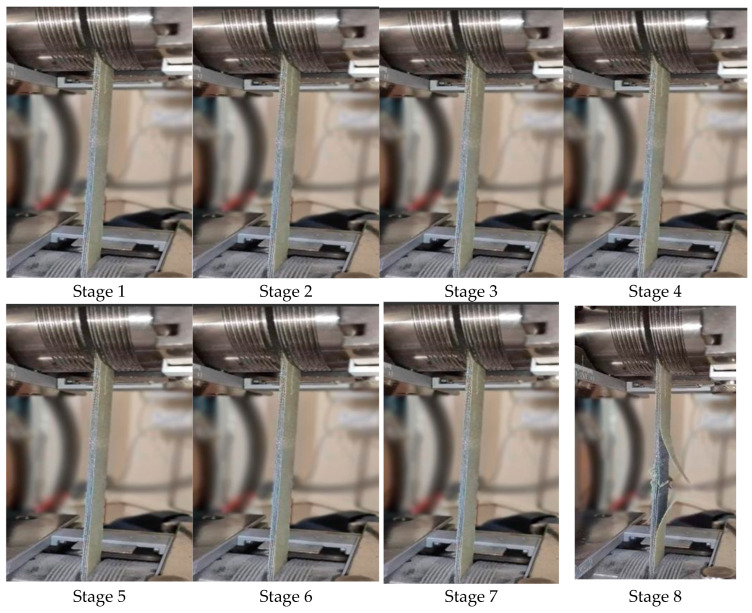
Various stages in the testing of the sample.

Upon testing the samples, the following results were achieved and compared with the results obtained when modeled and simulated. The step wise extension of the sample with the breaking of the sample in the final stage is shown in Figure 8.

The composite plates being utilized here were prepared according to the ASTM standards. The samples were then placed between the clamps of the tensile tester. The series of images shown in Figure 8 shows the various stages of extension of the sample as the test is being conducted and the last stage shows the end point of the test as the sample breaks. The series of images shown in Figure 8 is a general representation of the behavior of all of the samples.

### 3.1. Experimental Results

The following Table 3 shows the experimental results obtained after the tensile tests were conducted on the samples. The experiments were repeated 20 times for each sample and the mean values were reported. Further, the statistical significance was determined so as to have a minimum coefficient of variation (maximum CV% = 7.89%). Therefore, the experimental results are reliable, and the representativeness is authentic.

**Figure 9:**
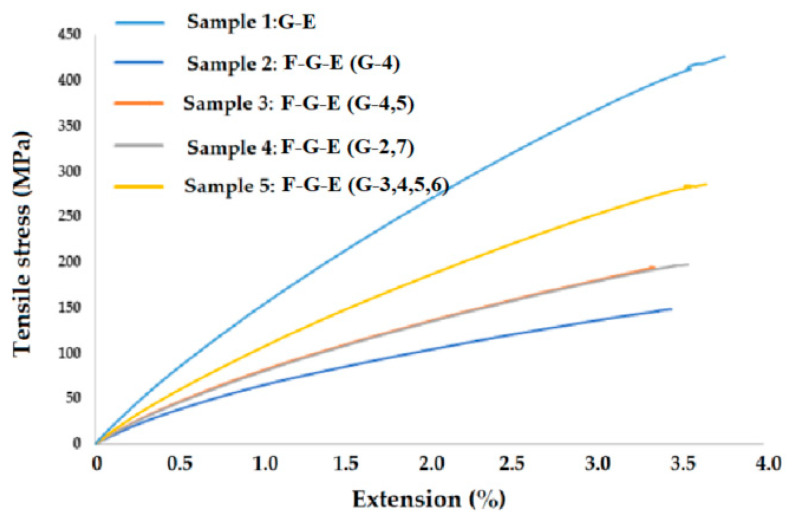
Graph showing tensile stress [MPa] v/s extension % for flax–glass–epoxy composites.

The tensile test results of the experimental samples are plotted in Figure 9.

From the results obtained, it can clearly be concluded that the tensile strength of the sample increases with the number of glass fiber layers. We can observe that the tensile strength of the samples with adjacent layers of glass fiber/fabric is higher than the samples with one layer of glass. On the other hand, when we compare the tensile strength of the samples with two layers of glass fabrics with the sample with four layers of glass fabrics, it can clearly be seen that the sample with the higher number of layers of glass fiber/fabrics resulted in higher tensile strength. The curves are almost linear, except for a kink in the end which can be due to fiber rupture or debonding between the fiber and matrix. Such observations are also found in the literature [43,44,45,46,47].

### 3.2. Simulation of Composite Samples

The simulation of different samples is shown in the following Figure 10, Figure 11, Figure 12, Figure 13, Figure 14, Figure 15, Figure 16, Figure 17, Figure 18, Figure 19, Figure 20, Figure 21, Figure 22 and Figure 23.

**Figure 10:**
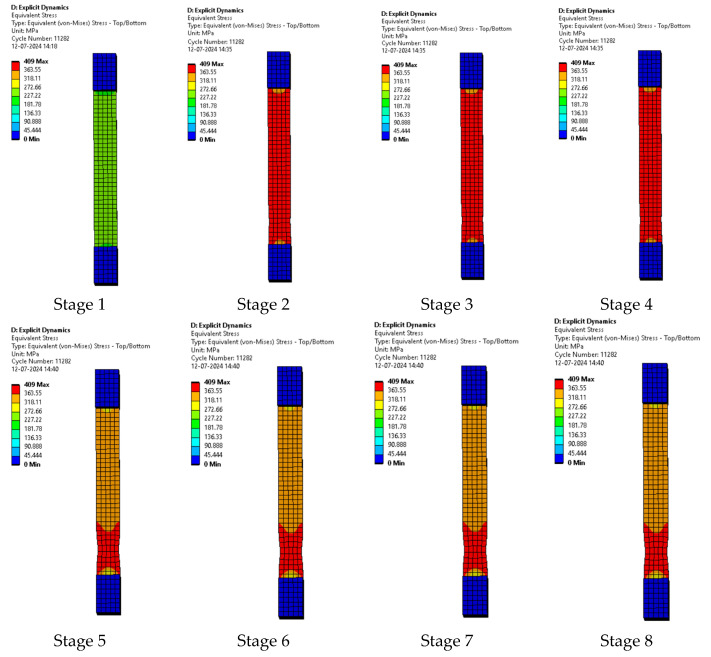
Various stages in the running of the simulation of sample 1: G-E.

#### 3.2.1. Sample 1: G-E

The series of images in Figure 10 represent the various stages of testing sample 1: G-E. While running the simulation for the samples, the first few images in the series represent the initial stages of simulation and the last few images represent the stages before the breaking of sample 1: G-E.

In the simulation, we can clearly make out the extension of the sample due to the changes in the color pattern of the elements that are being tested, whereas in the series of images shown in Figure 8, we can only make out the last stage (breakage) of the sample.

**Figure 11:**
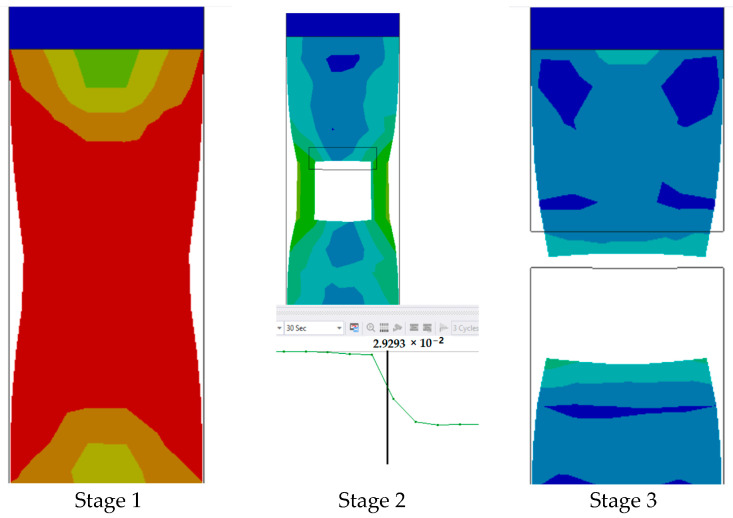
Stages showing the formation of breaks in sample 1: G-E.

The series of images shown in Figure 11 shows the stages in the formation of a break in sample 1: G-E and are a continuation of the series of images showing the various stages in the simulation of the samples shown in Figure 10. Here, in stage 1, we see the stage before the formation of the crack; in stage 2, we see the formation of the crack, and in stage 3, we see the total breakage of the sample.

**Figure 12:**
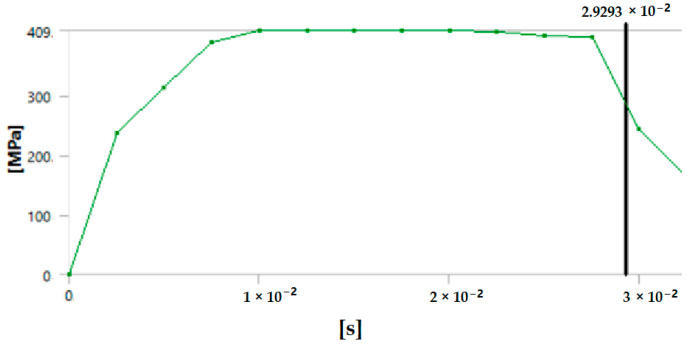
Graph showing the point of breaking of sample 1: G-E.

The graph in Figure 12 shows the time it takes for sample 1: G-E to break when the stress increases. Initially, the stress on the sample increases, and after the maximum level of stress is reached, the sample breaks. The breaking of the sample in the graph is observed when there is a sharp decrease in the stress.

Since the sample takes such a small amount of time to break, it is only possible to observe the breakage in the last stage, as shown in Figure 8 for all the samples. During the process of simulation, the process can be paused to observe the extension, with changes in the color of the elements of the sample, so it is easier to identify the various stages before the final breakage of the sample.

The graph shows the maximum value of stress at the time of breakage of the sample, as the percentage extension of the simulated sample was known via the physical testing of the sample and this value was used as one of the parameters while running the simulation to ascertain the value of stress required to the reach the calculated percentage extension value.

#### 3.2.2. Sample 2: F-G-E (G-4)

**Figure 13:**
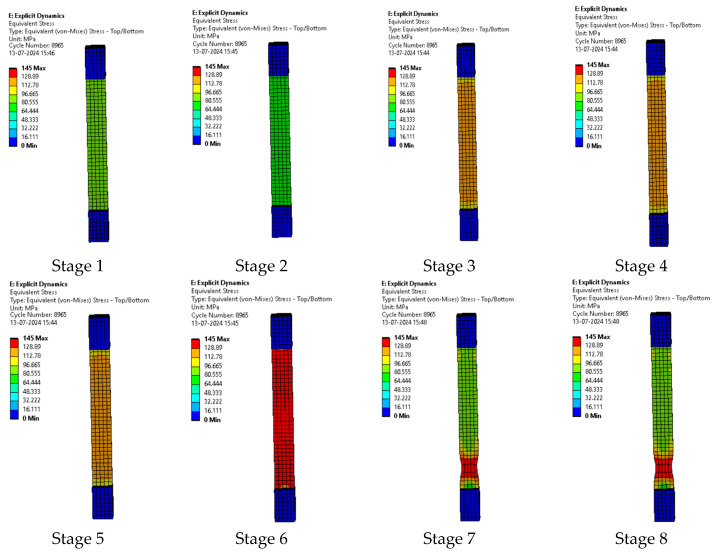
Various stages in the running of the simulation of sample 2: F-G-E (G-4).

The series of images in Figure 13 represent the various stages of testing sample 2: F-G-E (G-4). While running the simulation for sample 2: F-G-E (G-4), the first few images in the series represent the initial stages of simulation and the last few images represent the stages before the breaking of the sample.

In the simulation, we can clearly make out the extension of the sample due to the changes in the color pattern of the elements that are being tested, whereas in the series of images shown in Figure 8, we can only make out the last stage (breakage) of the sample.

**Figure 14:**
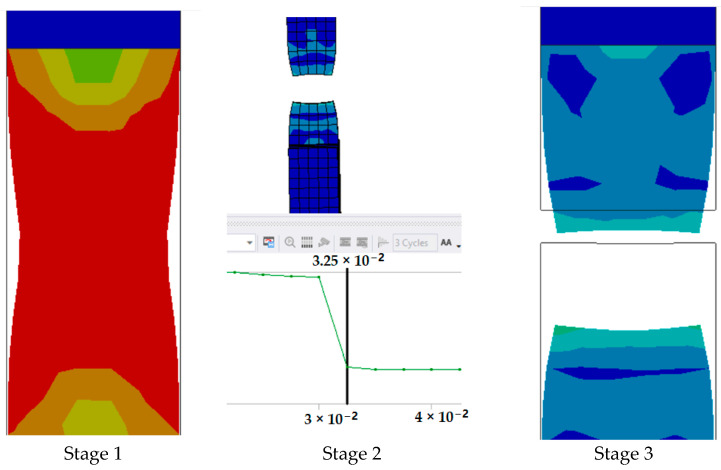
Stages showing the formation of breaks in sample 2: F-G-E (G-4).

The series of images shown in Figure 14 shows the stages in the formation of a break in sample 2: F-G-E (G-4) and are a continuation of the series of images showing the various stages in the simulation of the sample shown in Figure 13. Here, in stage 1, we the stage before the formation of the crack; in stage 2, we see the formation of a crack, and in stage 3, we see the total breakage of the sample.

**Figure 15:**
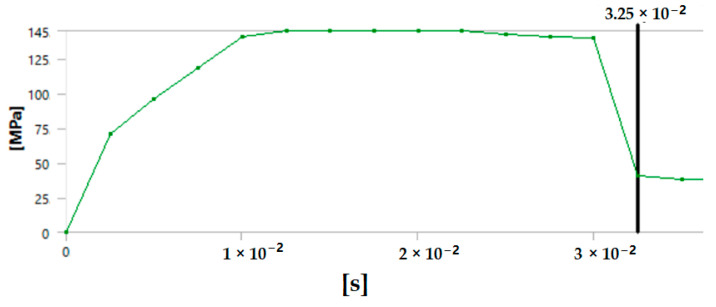
Graph showing the point of breaking of sample 2: F-G-E (G-4).

The graph in Figure 15 shows the time it takes for sample 2: F-G-E (G-4) to break when the stress increases. Initially, the stress on the sample increases, and after the maximum level of stress is reached, the sample breaks; the breaking of the sample is observed when there is a sharp decrease in the stress, as shown in the graph.

Since the sample takes such a small amount of time to break, it is only possible to observe the breakage in the last stage, as shown in Figure 8 for all the samples. During the process of simulation, the process can be paused to observe the extension, with changes in the color of the elements of the sample, so it is easier to identify the various stages before the final breakage of the sample.

The graph shows the maximum value of stress at the time of breakage of the sample, as the percentage extension of the simulated sample was known via the physical testing of the sample and this value was used as one of the parameters while running the simulation to ascertain the value of stress required to the reach the calculated percentage extension value.

#### 3.2.3. Sample 3: F-G-E (G-4,5)

**Figure 16:**
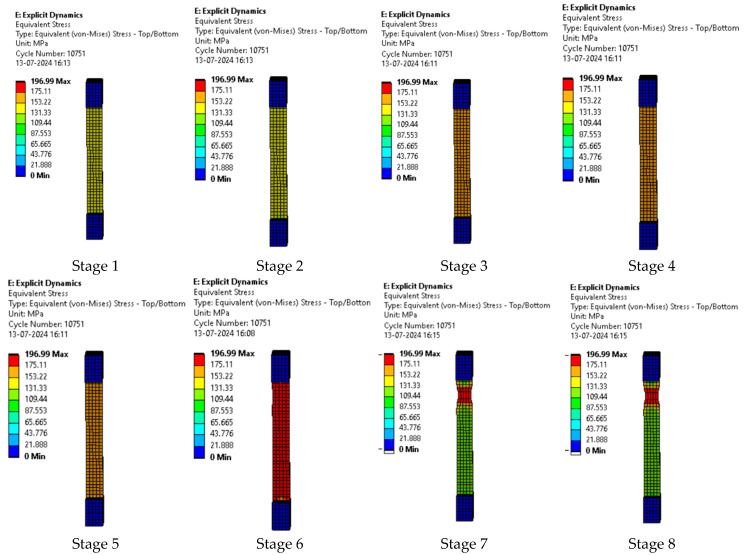
Various stages in the running of the simulation of sample 3: F- G-E (G-4,5).

The series of images in Figure 16 represent the various stages of testing of the sample 3: F-G-E (G-4,5). While running the simulation for the sample 3: F-G-E (G-4,5), the first few images in the series represents the initial stages of simulation and the last few images represent the stages before the breaking of the sample. In the simulation, we can clearly make out the extension of the sample due to the changes in the color pattern of the elements that are being tested, whereas in the series of images shown in Figure 8, we can only make out the last stage (breakage) of the sample.

**Figure 17:**
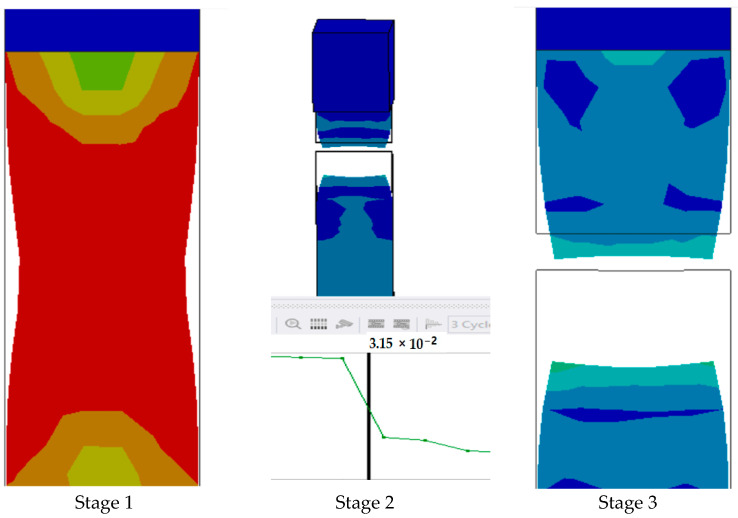
Stages showing the formation of breaks in sample 3: F- G-E (G-4,5).

The series of images shown in Figure 17 represent the stages in the formation of a break in sample 3: F-G-E (G-4,5) and are a continuation of the series of images showing the various stages in the simulation of the samples shown in Figure 16. Here, in stage 1, we see the stage before the formation of the crack; in stage 2, we see the formation of a crack, and in stage 3, we see the total breakage of the sample.

**Figure 18:**
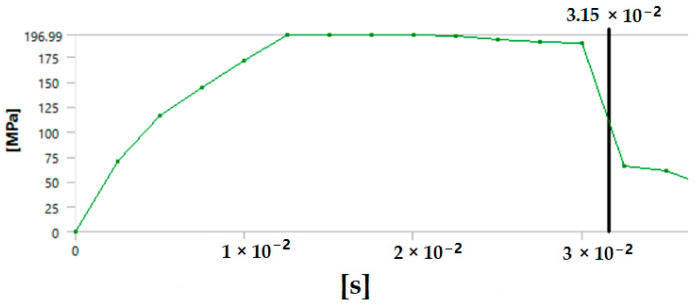
Graph showing the point of breaking of sample 3: F-G-E (G-4,5).

The graph in Figure 18 shows the time it takes for sample 3: F-G-E (G-4,5) to break when the stress increases. Initially, the stress on the sample increases, and after the maximum level of stress is reached, the sample breaks; the breaking of the sample is observed when there is a sharp decrease in the stress, as shown in the graph.

Since the sample takes such a small amount of time to break, it is only possible to observe the breakage in the last stage, as shown in Figure 8 for all the samples. During the process of simulation, the process can be paused to observe the extension, with changes in the color of the elements of the sample, so it is easier to identify the various stages before the final breakage of the sample.

The graph shows the maximum value of stress at the time of breakage of the sample, as the percentage extension of the simulated sample was known via the physical testing of the sample and this value was used as one of the parameters while running the simulation to ascertain the value of stress required to the reach the calculated percentage extension value.

#### 3.2.4. Sample 4: F-G-E (G-2,7)

**Figure 19:**
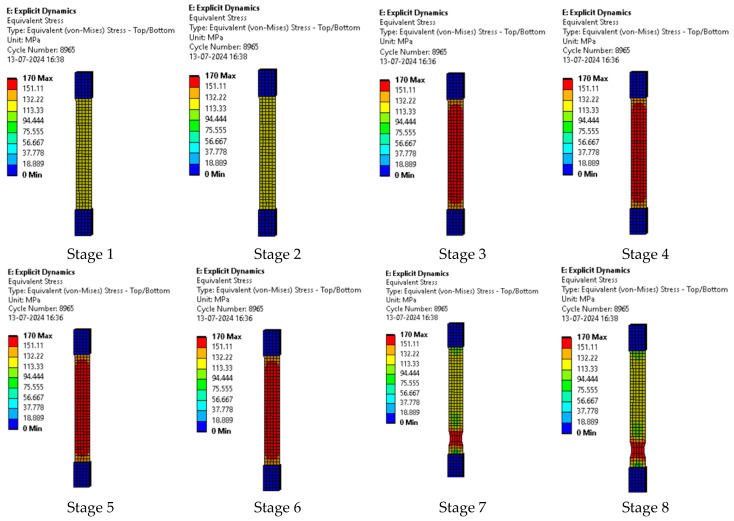
Various stages in the running of the simulation of sample 4: F-G-E (G-2,7).

The series of images in Figure 19 represent the various stages of testing of the sample 4: F-G-E (G-2,7). While running the simulation for the samples, the first few images in the series represent the initial stages of simulation and the last few images represent the stages before the breaking of the sample. In the simulation, we can clearly make out the extension of the sample due to the changes in the color pattern of the elements that are being tested, whereas in the series of images shown in Figure 8, we can only make out the last stage (breakage) of the sample.

**Figure 20:**
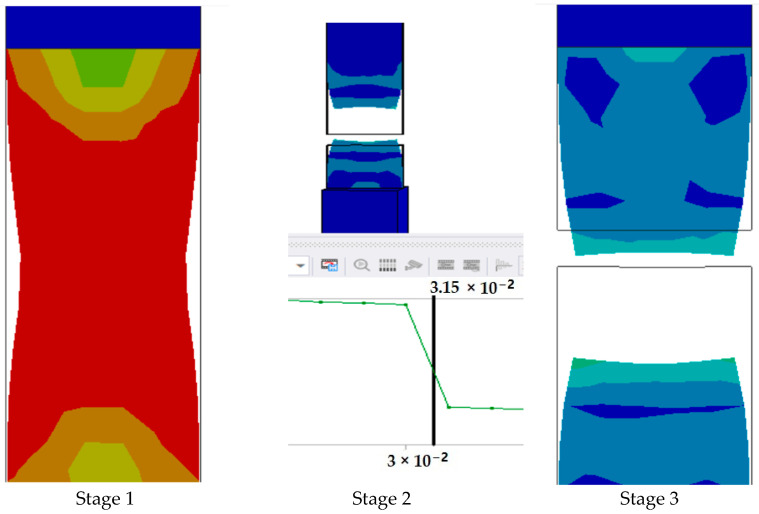
Stages showing the formation of breaks in sample 4: F-G-E (G-2,7).

The series of images shown in Figure 20 show the stages in the formation of a break in sample 4: F-G-E (G-2,7) and are a continuation of the series of images showing the various stages in the simulation of the samples shown in Figure 19. Here, in stage 1, we see the stage before the formation of the crack; in stage 2, we see the formation of a crack, and in stage 3, we see the total breakage of the sample.

**Figure 21:**
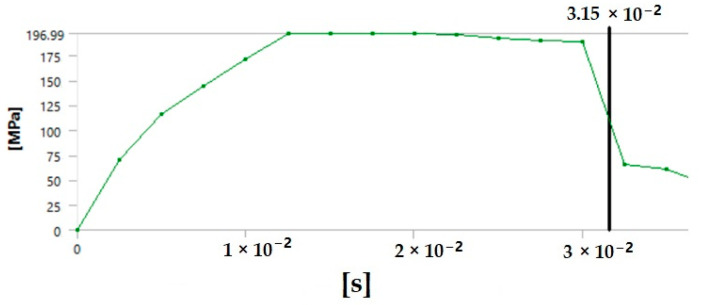
Graph showing the point of breaking of sample 4: F-G-E (G-2,7).

The graph in Figure 21 shows the time it takes for sample 4: F-G-E (G-2,7) to break when the stress increases. Initially, the stress on the sample increases, and after the maximum level of stress is reached, the sample breaks; the breaking of the sample is observed when there is a sharp decrease in the stress, as shown in the graph.

Since the sample takes such a small amount of time to break, it is only possible to observe the breakage in the last stage, as shown in Figure 8 for all the samples. During the process of simulation, the process can be paused to observe the extension, with changes in color of the elements of the sample, so it is easier to identify the various stages before the final breakage of the sample.

The graph shows the maximum value of stress at the time of breakage of the sample, as the percentage extension of the simulated sample was known via the physical testing of the sample and this value was used as one of the parameters while running the simulation to ascertain the value of stress required to the reach the calculated percentage extension value.

#### 3.2.5. Sample 5: F-G-E (G-3,4,5,6)

**Figure 22:**
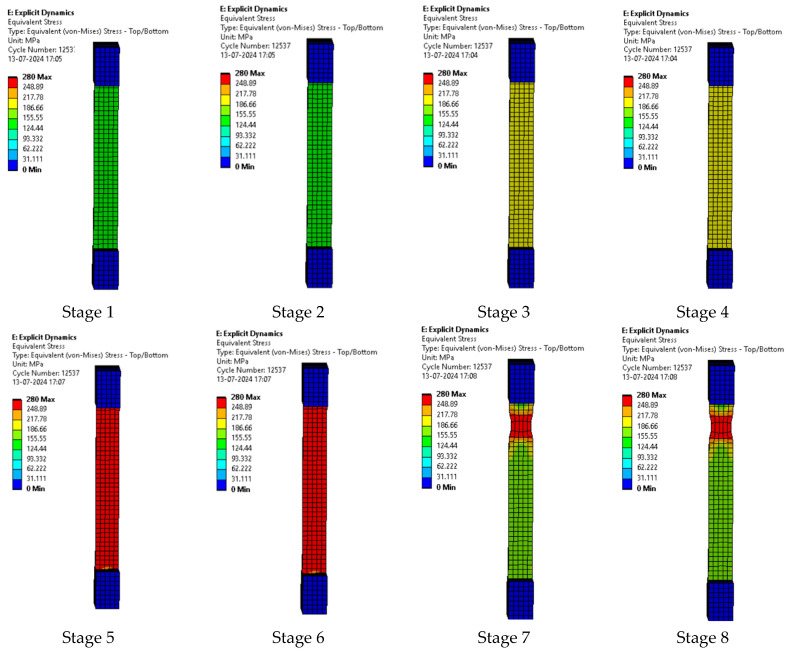
Various stages in the running of the simulation of sample 5: F-G-E (G-3,4,5,6).

The series of images in Figure 22 represent the various stages of testing of sample 5: F-G-E (G-3,4,5,6). While running the simulation for the samples, the first few images in the series represent the initial stages of simulation and the last few images represent the stages before the breaking of the sample. In the simulation, we can clearly make out the extension of the sample due to the changes in the color pattern of the elements that are being tested, whereas in the series of images shown in Figure 8, we can only make out the last stage (breakage) of the sample.

**Figure 23:**
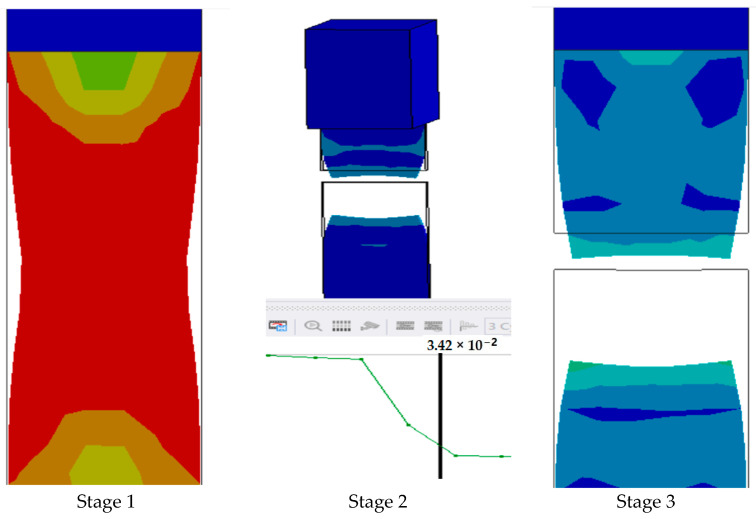
Stages showing the formation of breaks in sample 5: F-G-E (G-3,4,5,6).

The series of images shown in Figure 23 show the stages in the formation of a break in sample 5: F-G-E (G-3,4,5,6) and are a continuation of the series of images showing the various stages in the simulation of the samples shown in Figure 22. Here, in stage 1, we see the stage before the formation of the crack; in stage 2, we see the formation of a crack, and in stage 3, we see the total breakage of the sample.

**Figure 24:**
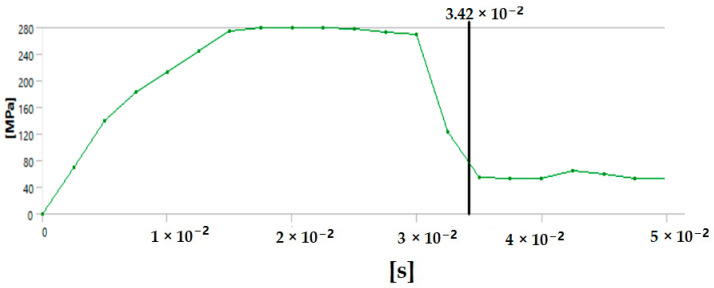
Graph showing the point of breaking of sample 5: F-G-E (G-3,4,5,6).

The graph in Figure 24 shows the time it takes for sample 5: F-G-E (G-3,4,5,6) to break when the stress increases. Initially, the stress on the sample increases, and after the maximum level of stress is reached, the sample breaks; the breaking of the sample is observed when there is a sharp decrease in the stress, as shown in the graph.

In the simulation, we can clearly make out the extension of the sample due to the changes in the color pattern of the elements that are being tested, whereas in the series of images shown in Figure 8, we can only make out the last stage (breakage) of the sample.

The graph shows the maximum value of stress at the time of breakage of the sample, as the percentage extension of the simulated sample was known via the physical testing of the sample and this value was used as one of the parameters while running the simulation to ascertain the value of stress required to the reach the calculated percentage extension value.

### 3.3. Validation of Simulation with Experimental Results

Upon testing the samples experimentally, the following results were obtained, and these were compared with the results obtained through simulation. The comparison is shown in Table 4 indicating the error %.

**Figure 25:**
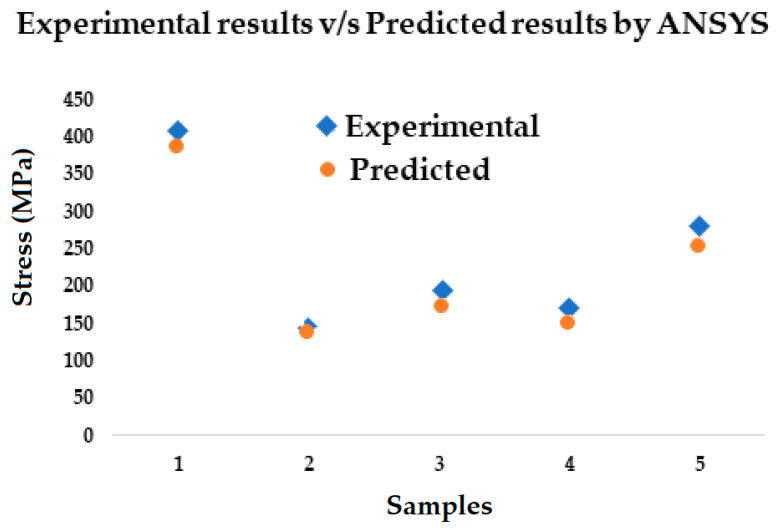
Graph showing tensile stress results of the composite samples [MPa], experimental v/s predicted by ANSYS for the simulated composite samples.

The comparison is also shown in Figure 25.

The results shown in Figure 25 are a comparison of the data obtained when the samples were tested physically and the data obtained when the material parameters were used to create the models and simulations were run. One can see that data obtained from the simulations are almost the similar to the data obtained from the physical tests.

Upon comparing the results obtained from testing the samples and the simulated results, we can see that the data obtained are almost the same as the data generated on ANSYS via simulations, and the error percentage varies from 4.05% to 8.05% for the various samples.

When we compare the tested samples, we observe that composites made from glass fiber and epoxy showed the maximum strength when compared to the samples which have layers of flax in their composition. It can be observed that the strength of the samples increases as the number of layers of glass fiber increases. This can be seen in both the physically tested samples and the samples simulated on ANSYS. Upon further observation, we can see that the strength of the composite with glass fiber as layers 4 and 5 has greater strength than the sample with glass fiber as layers 2 and 7, but the composite with glass fiber as layers 3, 4, 5 and 6 has greater strength than composites with two layers of glass fiber; this can also be observed in the samples that have been created and modeled in ANSYS.

### 3.4. Interlaminar Shear Strength (ILSS)

**Figure 26:**
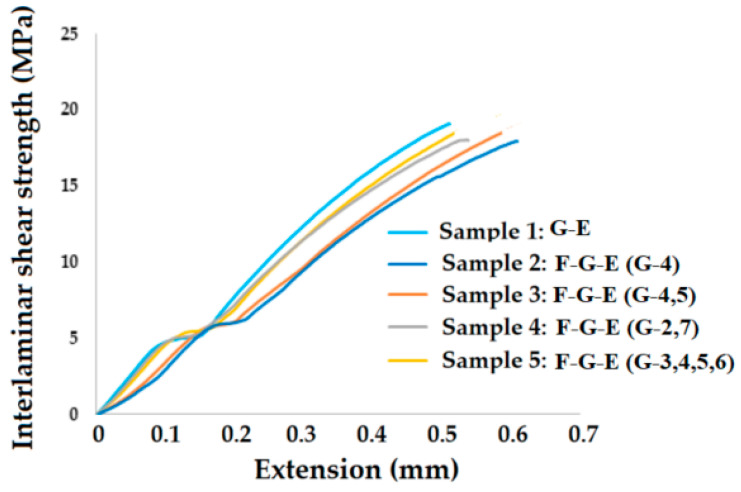
Interlaminar shear strength (ILSS) of the samples developed.

The interlaminar shear strength was measured as per ASTM D 2344 standard for all of the samples to further understand the bonding behavior between the layers of flax and glass fabrics. Also, the fiber–matrix bonding defines the ILSS. The results are shown in Figure 26.

The interlaminar shear strength shows a very similar trend as the tensile strength. This is because the principle of testing ILSS is based on a very short beam under 3-point bending. The 3-point bending involves an extension of the lower surface and longitudinal compression of the upper surface. Thus, both the surfaces undergo stress similar to tensile loading. This stress is gradually dissipated towards the core of the sample. At this point, interlayer or interlaminar bonding comes into picture. This ILSS is a result of fiber–matrix bonding and interlayer adhesion or cohesion between the fabric layers depending upon their composition or fiber type. In case of sample 1: G-E, it is a pure glass–epoxy composite without any flax fiber layers. Therefore, very strong interlaminar bonding exists between the glass fiber layers. Further, glass–epoxy bonding is also very strong, resulting in the maximum ILSS. This supports the observation of maximum tensile strength reported in this sample. Such observations are also found in the literature [48,49]. The minimum ILSS was observed for sample 2: F-G-E (G-4) because the four layers of glass make a group and are separated from the flax fabric layers. The inter glass fabric cohesion rather works negatively towards reducing the glass–flax bonding. These observations are supported by the results of tensile testing [34,35]. The second-best results were observed for sample 5: F-G-E (3,4,5,6). In this sample, the four glass layers form the core of the multilayered hybrid structure. The outer layers are formed by flax fibers. Thus, a strong consolidation of glass–epoxy results in the maximum tensile strength as well as shear strength. In the other samples with a relatively lower number of glass layers, the ILSS is also lower than the other samples, as observed in the trends in tensile testing [48,49,50].

### 3.5. Correlation of Tensile Strength and ILSS

**Figure 27:**
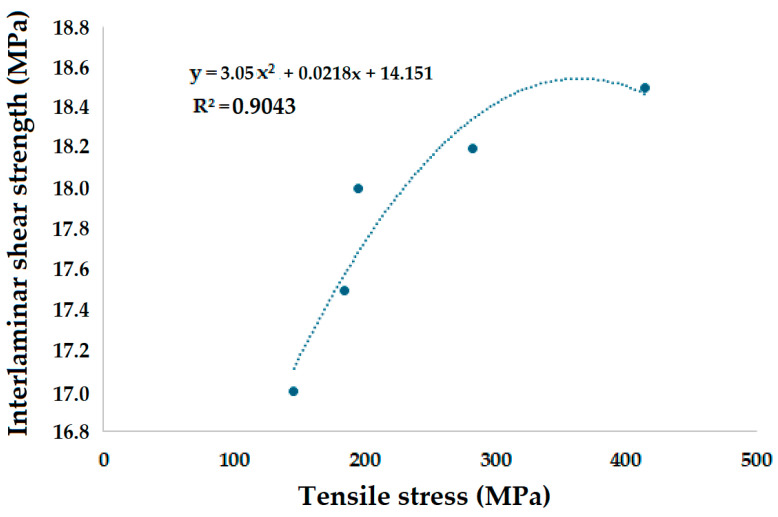
Correlation between interlaminar shear strength (ILSS) and tensile stress.

The results of tensile strength were correlated with that of interlaminar shear strength (ILSS) and plotted in Figure 27.

A very good correlation was observed between tensile stress and the interlaminar shear strength of the composite samples studied. A high coefficient of determination (R-square of 0.9043) was observed between these two types of mechanical performance.

### 3.6. Computed Tomography

**Figure 28:**
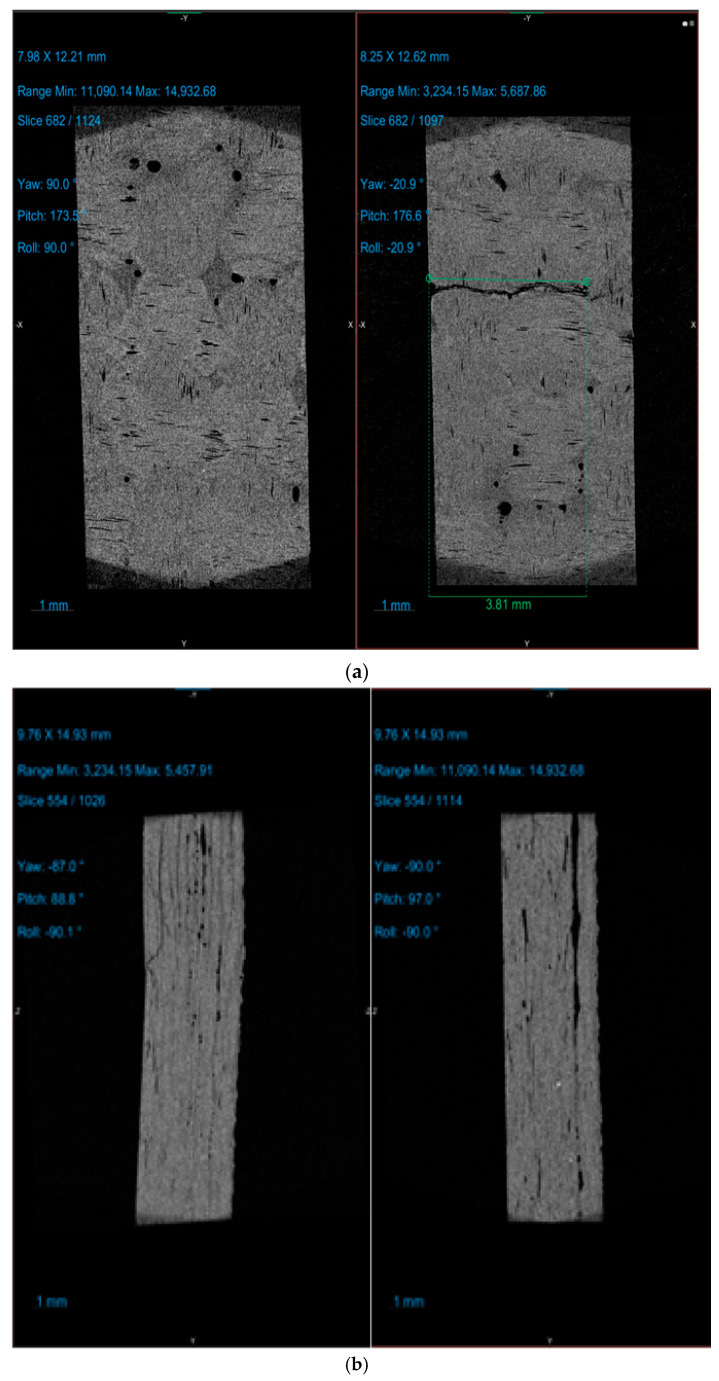

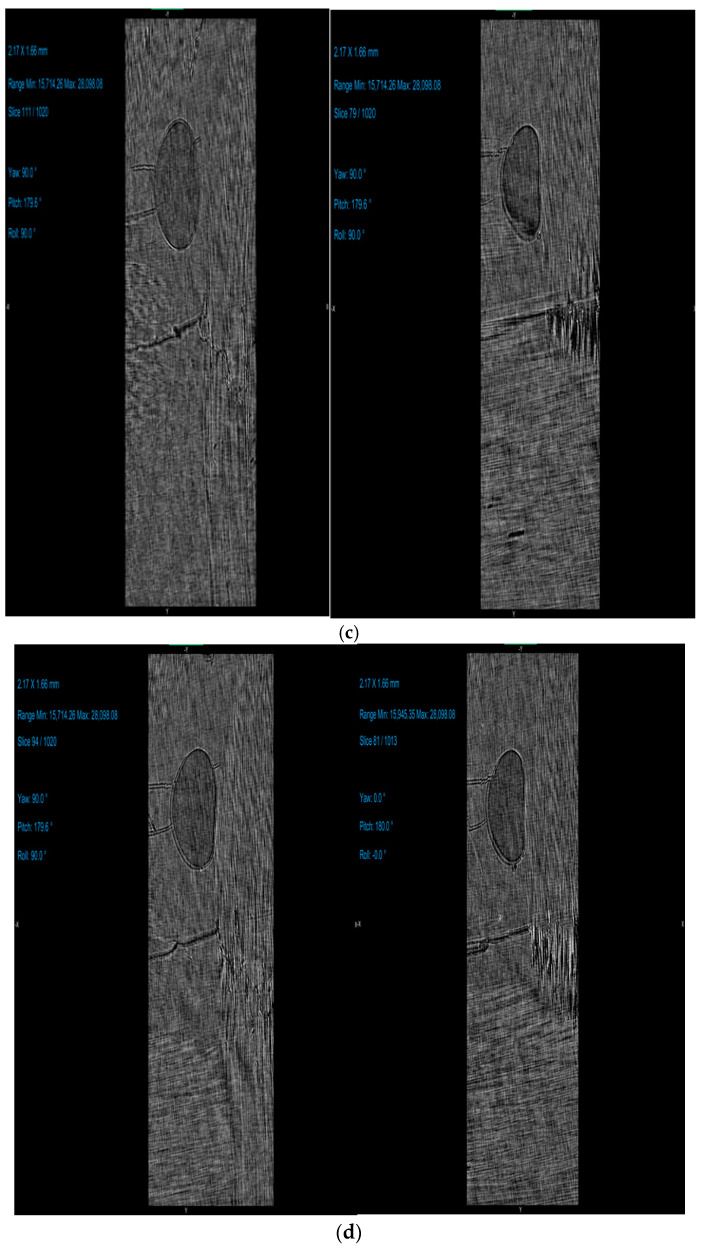

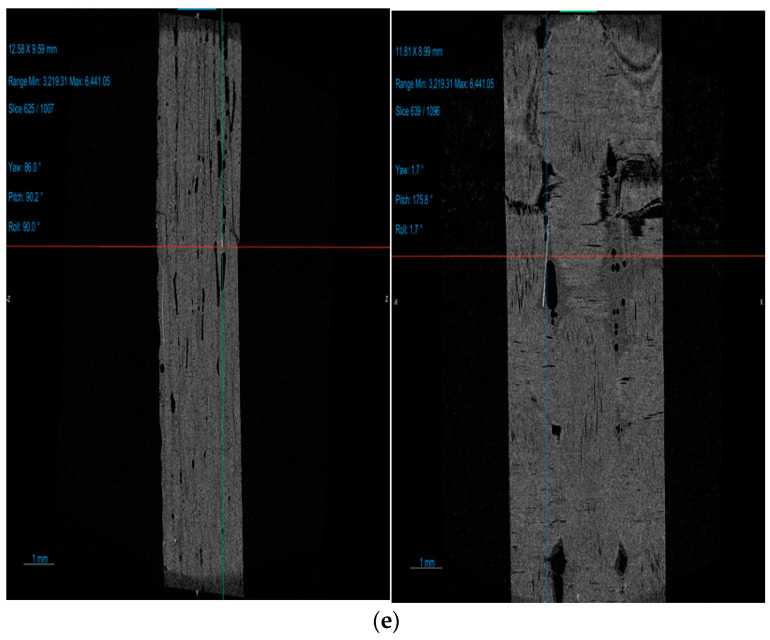
Computer tomography images of the samples before and after ILSS testing. (**a**) Sample 1: G-E. (**b**) Sample 2: F-G-E (G-4). (**c**) Sample 3: F- G-E (G-4,5). (**d**) Sample 4: F-G-E (G-2,7). (**e**) Sample 5: F-G-E (G-3,4,5,6).

Interlaminar cracks or fiber–matrix debonding was analyzed by using computer tomography as a tool. The CT scan images created from the scans are presented in Figure 28 for all the samples. The images show the internal structure before as well as after the ILSS tests.

The 3D CT scan images show the nature of fracture and failure modes of the different samples developed. Sample 1: G-E shows a cross-directional failure or cracking rather than interlaminar failure. This indicates strong fiber–matrix bonding and interlayer cohesion [51,52]. In the case of sample 2: F-G-E (G-4), interlaminar shearing or cracking is most prominent, indicating relatively poor adhesion between the core layers of glass and the outer layers of flax fabrics. The observations are also supported by the experimental results of tensile and ILSS testing. Sample 3: F-G-E (G-4,5) and sample 4: F-G-E (G-2,7) show similar failure modes or cracking behavior. These are combinations of cross-directional and longitudinal cracks. Due to the presence of several stress concentration points, the overall tensile strength and ILSS were intermediate [53,54]. Sample 5: F-G-E (G-3,4,5,6) shows very similar cracking behavior as sample 1 owing to the group of four glass layers in the core. However, multiple short cracks are generated between the glass and flax fabric layers. Overall, this sample stands second after the pure glass–epoxy composite sample. The results support the observations of tensile and ILSS testing [53,54].

## 4. Conclusions

Research was successfully conducted for determining the mechanical performance of glass–flax–epoxy hybrid composites using ANSYS-based computational models. Data obtained after simulation and experimental testing of the samplers revealed that the higher the number of layers of glass fabrics, the higher the strength of the composite. This was observed after comparing the results of the tensile tests performed on composite samples with four layers of glass fabrics with those of the samples with two layers of glass fabrics. On the other hand, when we compared the samples which had eight layers of glass fabrics with the samples with four layers of glass and four layers of flax, the sample with eight glass fabric layers exhibited greater strength. The maximum tensile strength of 415 MPa was found for sample 1: G-E and the minimum strength of 146 MPa was found for sample 2: F-G-E (G-4). Further, when we compared the samples which had two layers of glass, it was found that the sample with the glass fiber on the fourth and fifth layers exhibited greater strength than the sample which had glass fibers as the second and seventh layers. These observations were consistent both with the ANSYS-based simulation and the experimental testing. When the data obtained from the simulations were compared to the data obtained from the physical tests, an error percentage which varied from 4.05% to 8.05% was found.

The experimental study of the tensile properties of polymer matrix composites was further supplemented with interlaminar shear strength (ILSS) tests, and a high accuracy (R-square of 0.9043) was found. The maximum interlaminar shear strength (ILSS) of 18.5 MPa was observed for sample 1: G-E and the minimum ILSS of 17.0 MPa was observed for sample 2: F-G-E (G-4). Computer tomography (CT-An) enabled us to analyze internal fractures during the ILSS testing. Sample 2: F-G-E (G-4) showed significantly visible interlaminar cracking between the layers of glass and flax at the interface. Sample 1: G-E showed a failure through the sample cross section rather than interlaminar failure, which was attributed to stronger fiber–matrix bonding and interlayer cohesion.

It can be concluded that for applications which require greater tensile and interlaminar shear strength, hybrid composites with a higher number of high-strength fibers should be used in combination with a lower number of natural fiber layers. Further, the performance can be determined based on the sequential arrangements of the different types of fibers in the layers. The best possible combination of layers and their arrangement is subject to further investigation in the future. The results are useful for developing light weight hybrid composite materials which can replace specific components in the construction of vehicles in the future.

## Figures and Tables

**Table 1:** Properties of the fiber/fabric materials used [35,36,37,38,39,40].

Properties	Glass	Flax
Fiber fineness/diameter (µm)	21 ± 1.1	20 ± 1.2
Fiber linear density (Tex, g/km)	20 ± 1.1	21 ± 1.2
Fiber average length (mm)	4–6	4–8
Density (g/cm^3^)	2.48 ± 0.2	1.5 ± 0.1
Young’s modulus of tow/yarn (GPa)	86.5 ± 1.4	37.5 ± 0.8
Bulk modulus for tow/yarn (GPa)	37.7 ± 1.5	15.4 ± 1.1
Strength (GPa)	4.65 ± 0.15	2.47 ± 0.05
Tow/yarn linear density (Tex, g/km)	600 ± 2	600 ± 11
Fabric areal density (g/m^2^)	600 ± 10	600 ± 25
Warp density in fabric (cm^−1^)	15	15
Weft density in fabric (cm^−1^)	14	14

**Table 2:** List of samples developed.

Scheme	Sample	Sample Code	Orientation	Number of Layers	Layers							
					1	2	3	4	5	6	7	8
1	Glass–epoxy	G-E	0/90	8	G	G	G	G	G	G	G	G
2	Flax–glass–epoxy	F-G-E	0/90	8	F	F	F	G	F	F	F	F
3	Flax–glass–epoxy	F-G-E	0/90	8	F	F	F	G	G	F	F	F
4	Flax–glass–epoxy	F-G-E	0/90	8	F	G	F	F	F	F	G	F
5	Flax–glass–epoxy	F-G-E	0/90	8	F	F	G	G	G	G	F	F

G—glass, F—flax.

**Table 3:** Results of tensile test.

Sl. No.	Sample Details	σ_max_ (MPa)			Ɛ_max_ Force (%)			Number of Specimens Tested
		Mean	SD	CV (%)	Mean	SD	CV (%)	
1	G-E	414.0	7.40	1.78	3.6	0.1	2.56	5
2	F-G-E (G-4)	145.0	1.16	0.80	3.4	0.0	1.26	5
3	F-G-E (G-4,5)	195.0	1.14	0.58	3.5	0.1	1.45	5
4	F-G-E (G-2,7)	181.0	9.41	5.08	3.1	0.2	7.89	5
5	F-G-E (G-3,4,5,6)	285.0	2.48	0.88	3.6	0.1	1.91	5

**Table 4:** Comparison between test results obtained and predicted results.

Samples	Number of Layers	Extension %	Test Results (MPa)	Predicted Results (MPa)	Error %
G-E	8	3.6	414.0	388.4	6.1%
F-G-E(G-4)	8	3.4	145.0	137.7	5.03%
F-G-E(G-4,5)	8	3.5	195.0	187.8	3.69%
F-G-E(G-2,7)	8	3.1	181.0	177.5	3.5%
F-G-E(G-3,4,5,6)	8	3.6	285.0	260.2	8.7%

F—flax fiber, E—epoxy resin, G—glass fiber.

## Data Availability

The original contributions presented in the study are included in the article. Further inquiries can be directed to the corresponding author.
